# JmjC-KDMs KDM3A and KDM6B modulate radioresistance under hypoxic conditions in esophageal squamous cell carcinoma

**DOI:** 10.1038/s41419-020-03279-y

**Published:** 2020-12-14

**Authors:** Catarina Macedo-Silva, Vera Miranda-Gonçalves, Ana Lameirinhas, Joana Lencart, Alexandre Pereira, João Lobo, Rita Guimarães, Ana Teresa Martins, Rui Henrique, Isabel Bravo, Carmen Jerónimo

**Affiliations:** 1 Cancer Biology & Epigenetics Group – Research Center, Portuguese Oncology Institute of Porto (CI-IPOP), Porto, Portugal; 2 Medical Physics, Radiobiology and Radiation Protection Group - Research Center, Portuguese Oncology Institute of Porto (CI-IPOP), Porto, Portugal; 3 Departments of Medical Physics, Portuguese Oncology Institute of Porto, Porto, Portugal; 4 Departments of Pathology, Portuguese Oncology Institute of Porto, Porto, Portugal; 5 Department of Pathology and Molecular Immunology, Institute of Biomedical Sciences Abel Salazar – University of Porto (ICBAS-UP), Porto, Portugal

**Keywords:** Predictive markers, Cancer

## Abstract

Esophageal squamous cell carcinoma (ESCC), the most frequent esophageal cancer (EC) subtype, entails dismal prognosis. Hypoxia, a common feature of advanced ESCC, is involved in resistance to radiotherapy (RT). RT response in hypoxia might be modulated through epigenetic mechanisms, constituting novel targets to improve patient outcome. Post-translational methylation in histone can be partially modulated by histone lysine demethylases (KDMs), which specifically removes methyl groups in certain lysine residues. KDMs deregulation was associated with tumor aggressiveness and therapy failure. Thus, we sought to unveil the role of Jumonji C domain histone lysine demethylases (JmjC-KDMs) in ESCC radioresistance acquisition. The effectiveness of RT upon ESCC cells under hypoxic conditions was assessed by colony formation assay. KDM3A/KDM6B expression, and respective H3K9me2 and H3K27me3 target marks, were evaluated by RT-qPCR, Western blot, and immunofluorescence. Effect of JmjC-KDM inhibitor IOX1, as well as *KDM3A* knockdown, in in vitro functional cell behavior and RT response was assessed in ESCC under hypoxic conditions. In vivo effect of combined IOX1 and ionizing radiation treatment was evaluated in ESCC cells using CAM assay. KDM3A, KDM6B, HIF-1α, and CAIX immunoexpression was assessed in primary ESCC and normal esophagus. Herein, we found that hypoxia promoted ESCC radioresistance through increased KDM3A/KDM6B expression, enhancing cell survival and migration and decreasing DNA damage and apoptosis, in vitro. Exposure to IOX1 reverted these features, increasing ESCC radiosensitivity and decreasing ESCC microtumors size, in vivo. KDM3A was upregulated in ESCC tissues compared to the normal esophagus, associating and colocalizing with hypoxic markers (HIF-1α and CAIX). Therefore, KDM3A upregulation in ESCC cell lines and primary tumors associated with hypoxia, playing a critical role in EC aggressiveness and radioresistance. KDM3A targeting, concomitant with conventional RT, constitutes a promising strategy to improve ESCC patients’ survival.

## Introduction

Esophageal cancer (EC) is the eighth most common cancer worldwide and the sixth most common cause of death from cancer^1,2^. Esophageal squamous cell carcinoma (ESCC) is the most common histological subtype^3–5^. Although most patients are diagnosed with a loco-regional disease, surgery remains the cornerstone of curative-intent treatment, despite the high morbidity and mortality rates^6,7^. Indeed, overall 5-year survival rates do not exceed 15–20%^8^. In addition to surgery, radiotherapy (RT) is often used as the first-line treatment of EC, both as the main therapeutic strategy or in neoadjuvant context, combined with chemotherapy, entailing similar survival rates in advanced ESSC^7,9^.

Cellular hypoxia is a common feature of most solid tumors, including EC, and it has been related to therapy resistance^10,11^. Indeed, in oxygen deprived microenvironment, ionizing radiation (IR) has less impact on DNA damage, once is more efficiently repaired^12^. Hypoxia-inducible factors (HIFs) mediates tumor cells’ adaptation to hypoxic microenvironment. Specifically, the HIF transcription factor is a heterodimer composed of an oxygen-dependent α subunit and by a constitutively expressed non-oxygen dependent β subunit. The α subunit was reported to be degraded in the presence of oxygen (>5% O_2_). Furthermore, is translocated to the nucleus to form the HIF–α/β complex, which binds to specific promoter regions in hypoxia-responsive elements (HREs). Binding of HIF–α/β to HRE results in transcriptional upregulation of target genes typically involved in cell survival, cell metabolism, proliferation, and angiogenesis^13^. Interestingly, in EC, HIF-1α upregulation is considered a promising endogenous hypoxia biomarker^14^ and HIF-1α-mediated upregulation of carbonic anhydrase IX (CAIX) associates with poor prognosis^11,15–19^.

Hypoxia also modulates the epigenetic landscape, affecting DNA methylation and enzymes responsible for histone post-translational modifications^11,20–22^. Among the latter, histone lysine demethylases (KDM) of Jumonji C domain family (JmjC-KDMs), exhibit different methylation and histone substrates specificity^23^. JmjC-KDMs activity is dependent on oxygen and 2-oxoglutarate availability, as substrates^24^. Interestingly, JmjC-KDMs are modulated by hypoxia, through altered activity or expression levels (a direct mechanism) or through HIF1-mediation (an indirect mechanism)^23,25,26^. Although several KDM subfamilies have been found upregulated in hypoxia, only some of them are direct targets of HIF-1^23^. In particular, KDM3A and KDM6B are activated in hypoxic tumors and associated with tumor aggressiveness and progression^23,27^. Furthermore, these same enzymes have been suggested as promising therapeutic targets in cancer^28–30^. Thus, we sought to characterize the role of JmjC-KDMs activity in ESCC radioresistance, under hypoxia, aiming to improve the effectiveness of RT through JmjC-KDMs modulation.

## Results

### Hypoxia decreases RT response in ESCC

In both in vitro hypoxic conditions, HIF-1α chemical induction with 50 µM CoCl_2_ or 0.5-1% of O_2_ levels, nuclear HIF-1α and cell membrane CAIX expression were increased in all cell lines, although a more impressive effect was observed in Kyse-30 and OE21 cells [which did not express these proteins in normoxia (21% O_2_ levels)] (Supplementary Fig. S1A, B).

**Fig. 1:**
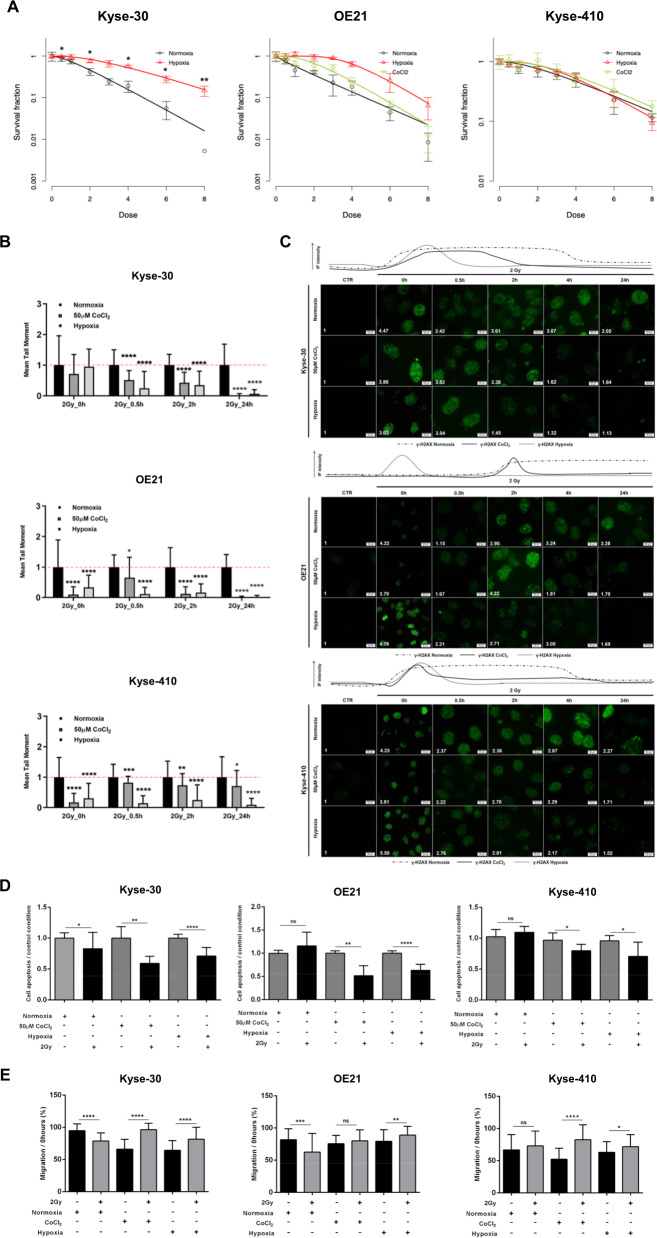
Effect of hypoxia on RT response, DNA damage, cell migration, and apoptosis. **a** Cell surviving fraction in three ESCC cell lines irradiated with [0–8] Gy range concentration under normoxia, 50 µM CoCl_2_ and hypoxia through SHMT model analysis. Results are presented as mean±SD of at least 3 independent experiments. **b** DNA damage of 2 Gy irradiated ESCC cells between 0 and 24h, under normoxia, 50 µM CoCl_2_ and hypoxia by comet assay. The results are the mean of at least 50 comets per condition. All values of DNA fragmentation (tail moment) were normalized to control (0Gy). Further, hypoxic conditions (50µM CoCl_2_ or 0.5–1% O_2_) were compared to normoxia. **c** Representative pictures of nuclear γ-H2AX staining of 2 Gy irradiated ESCC cells under normoxia, 50 µM CoCl_2_ and hypoxia conditions. All pictures were taken from Olympus IX51 microscope at ×400 magnification (scale bar 20 μm). IF quantification was done using ImageJ software (version 1.6.1, from National Institutes of Health) and represented as a fold change between 2 Gy irradiated cells and non-irradiated control. IF, fluorescence intensity. **d** ESCC cell apoptosis under normoxia, 50 µM CoCl_2_ and hypoxia conditions with 2 Gy of IR. Results are presented as mean±SD of at least three independent experiments. **e** ESCC cell migration through wound-healing assay, after 24 h of 2 Gy treatment normalized 0 h. Results are the mean±SD of at least three independent experiments; Irradiated cells are compared to non-irradiated cells in each condition. *p < 0.05; **p < 0.01; ***p < 0.001; ****p < 0.0001.

Kyse-30 and OE21 cells survival fraction were increased in both hypoxic conditions, whereas no effect was apparent for Kyse-410, since a radioresistant behavior comparing with the other ones (Fig. 1a and Supplementary Fig. S1C). Furthermore, D_0_, D_q_, and SF2 values were found increased in hypoxia (50 µM CoCl_2_ or 0.5-1% O_2_ levels) compared with normoxia in Kyse-30 and OE21 cell lines (Supplementary Table S1). To determine whether hypoxia might regulate double strand breaks (DSB) repair, γ-H2AX foci staining and DNA fragmentation were assessed through immunofluorescence (IF) and comet assay, respectively. Indeed, global DNA fragmentation was significantly decreased up to 24 h after hypoxic-induced ESCC cell lines irradiation (2 Gy) (Fig. 1b and Supplementary Fig. S2a). Moreover, both normoxia and hypoxia conditions presented γ-H2AX foci at 30 min, although ESCC cells in hypoxia (50 µM CoCl_2_ or 0.5-1% O_2_ levels) showed a notable decrease over the time (Fig. 1c), indicating lesser DNA damage and higher radioresistance. In addition, decreased apoptosis was observed for all ESCC cell lines after 24 h irradiation in hypoxia (50 µM CoCl_2_ or 0.5-1% of O_2_ levels) comparing with normoxia (Fig. 1d). Contrarily, cell migration capacity increased in irradiated ESCC cells under hypoxic conditions, except for Kyse-410 treated with 50 µM CoCl_2_ (Fig. 1e).

### Hypoxia modulates KDM3A and KDM6B expression in ESCC

**Fig. 2:**
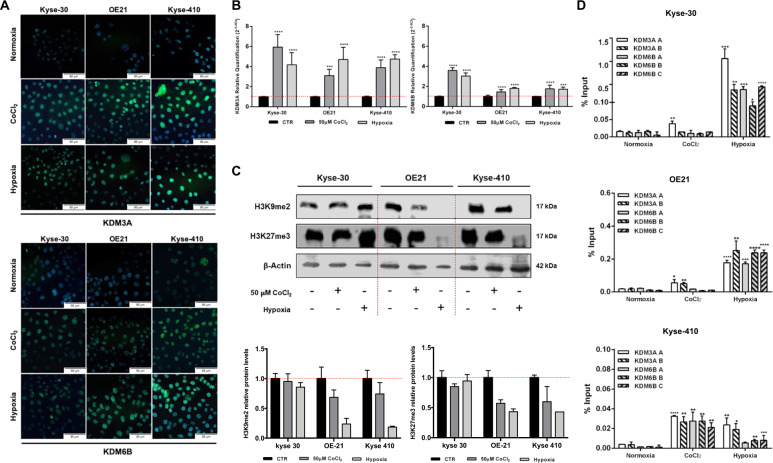
JmjC-KDMs expression in ESCC cells under hypoxic conditions. **a** Nuclear KDM3A and KDM6B expression in normoxia and hypoxic (50 µM CoCl_2_ or 0.5–1% O_2_) conditions, by IF. All pictures are taken from Olympus IX51 microscope at ×200 magnification (scale bar 50 μm). **b** KDM3A and KDM6B transcript levels under normoxia and hypoxic (50 µM CoCl_2_ or 0.5–1% O_2_) conditions. Results represent the mean ± SD of at least three independent replicates, each one in triplicate; ***p < 0.001; ****p < 0.0001. Hypoxic conditions were compared to normoxia. **c** Representative images of total protein levels of H3K9me2 (17 kDa), H3K27me3 (17 kDa) under normoxia and hypoxic (50 µM CoCl_2_ or 0.5–1% O_2_) conditions. β-actin (42 kDa) was used as loading control. **d** HIF-1α binding at KDM3A and KDM6B promoter region in normoxia and hypoxic (50 µM CoCl_2_ or 0.5–1% O_2_) conditions through ChIP assay. Values of 0.5–1% O_2_ and 50 µM CoCl_2_ were normalized to normoxia; *p < 0.05; **p < 0.01; ***p < 0.001; ****p < 0.0001.

Remarkably, in hypoxia (50 µM CoCl_2_ or 0.5–1% of O_2_ levels), ESCC cells disclosed higher KDM3A and KDM6B nuclear protein expression, as well as transcript levels (Fig. 2a, b). Conversely, H3K9me2 and H3K27me3, repressive histone markers^31^ targeted by KDM3A and KDM6B, respectively, were found downregulated under hypoxic conditions, except for Kyse-30 (Fig. 2c). Moreover, significant HIF-1α binding to tested promoter regions of KDM3A and KDM6B was observed under hypoxia conditions, corroborating HIF-1α-JmjC-KDMs interaction (Fig. 2d). Nonetheless, the same was not observed for KDM6B in Kyse-30 and OE21 cells exposed to 50 µM CoCl_2_ (Fig. 2d).

### IOX1 and KDM3A knockdown increases ESCC RT response

**Fig. 3:**
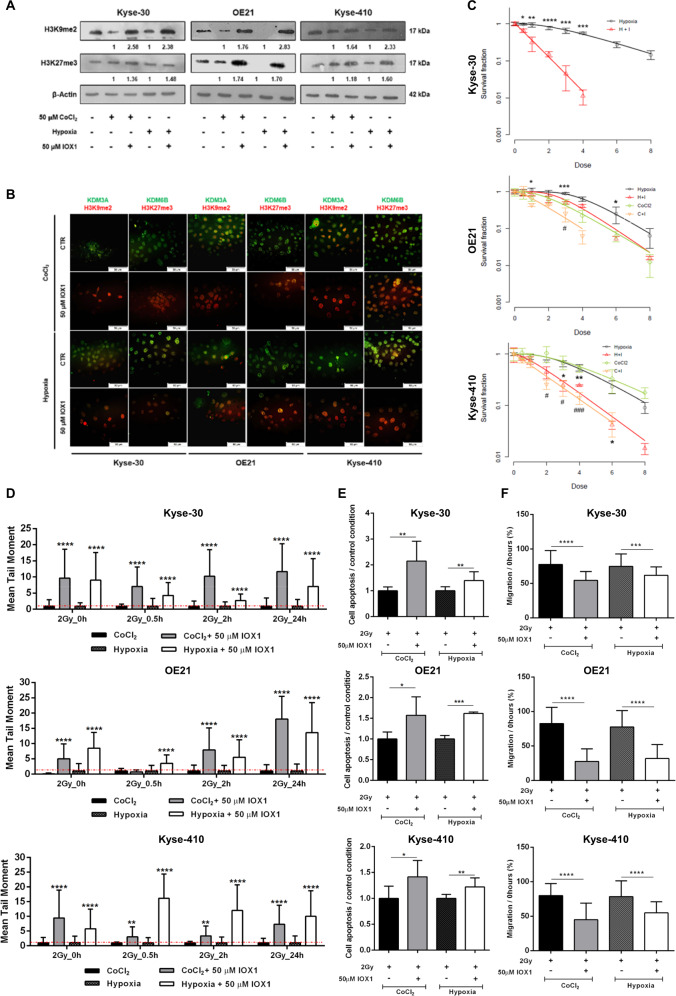
JmjC-KDMs activity inhibition effect on RT response in ESCC hypoxic cell lines. **a** Representative images of total protein levels of H3K9me2 (17 kDa), H3K27me3 (17 kDa) with 50 μM IOX1 JmjC-KDM inhibitor under hypoxic conditions (50 µM CoCl_2_ or 0–1% O_2_). β-actin was used as loading control. CoCl_2_ + 50 μM IOX1 and hypoxia + 50 μM IOX1 were normalized to CoCl_2_ and hypoxia alone, respectively. A representative picture of three independent replicates was chosen. **b** Representative merged staining pictures of KDM3A and KDM6B (green fluorescence) with H3K9me2 and H3K27me3 (red fluorescence). All pictures were taken from Olympus IX51 microscope at ×200 magnification (scale bar 50 μm). **c** Cell survival curves for 20 μM IOX1 in combination with irradiation treatment [0–8 Gy] under hypoxic conditions (50 µM CoCl_2_ and 0–1% O_2_). Results are presented as mean±SD of at least three independent experiments; (*) comparison between 20 μM IOX1 + hypoxia and hypoxia alone; (#) comparison between 20 μM IOX1 + 50 µM CoCl_2_ and 50 µM CoCl_2_ alone; *p < 0.05; **p < 0.01; ***p < 0.001; *p* < 0.0001; #*p* < 0.05; ###*p* < 0.001. **d** Effect of combined 50 µM IOX1 + 2 Gy irradiation treatment in DNA damage of ESCC cells for 50 µM CoCl_2_ and hypoxia conditions by comet assay. The results are the mean of at least 50 comets per condition. Results were normalized to 0 Gy and combined treatment at hypoxic conditions (50 µM CoCl_2_ or 0–1% O_2_) were compared to hypoxic condition alone; ****p < 0.0001. **e** Cell apoptosis and (**f**) Cell migration, for combined 50 µM IOX1 + 2 Gy irradiation treatment under hypoxic conditions (50 µM CoCl_2_ or 0–1% O_2_). Hypoxic conditions (50 µM CoCl_2_ or 0–1% O_2_) with IOX1 were compared to hypoxic conditions alone. Results are represented as mean ± SD of at least three independent experiments; *p < 0.05; **p < 0.01; ***p < 0.001; ****p < 0.0001.

ESCC cells treatment with 50 µM of IOX1 increased H3K9me2 and slightly alter H3K27me3 levels (Fig. 3a, b), whereas decreased protein expression of KDM3A and KDM6B was also apparent in both in vitro hypoxic conditions (Fig. 3b).

Moreover, KDMs activity inhibition with 20 µM IOX1, under 50 µM CoCl_2_ or 0.5–1% of O_2_ levels, induces decreased cell survival fraction in all ESCC cells (Fig. 3c). Concomitantly, D_0_, D_q_ and SF2 values were decreased in hypoxia (50 µM CoCl_2_ or 0.5–1% of O_2_ levels) combined with IOX1, comparatively with hypoxic conditions alone (Supplementary Table S2). Interestingly, IOX1 sensitized hypoxic ESCC cells to RT [SER > 1]. Specifically, Kyse-30 under hypoxia (0.5–1% O_2_ levels) disclosed the highest sensitization values with IOX1 treatment (SER = 3.89) (Supplementary Table S2).

Remarkably, for all ESCC cells, IOX1 treatment combined with 2 Gy irradiation significantly increased the % of global DNA fragmentation and cell apoptosis for most of the time points, compared to 50 µM CoCl_2_ and 0.5–1% O_2_ conditions (Fig. 3d, e and Supplementary Fig. S2B). Furthermore, cell migration capability was significantly decreased in IOX1 treated cells (Fig. 3f). Of note, 50 µM IOX1 effect was significantly lower in normal esophageal Het-1A cell line than in ESCC cell lines (Supplementary Fig. S3), both for cell viability and apoptosis (Supplementary Fig. S3).

**Fig. 4:**
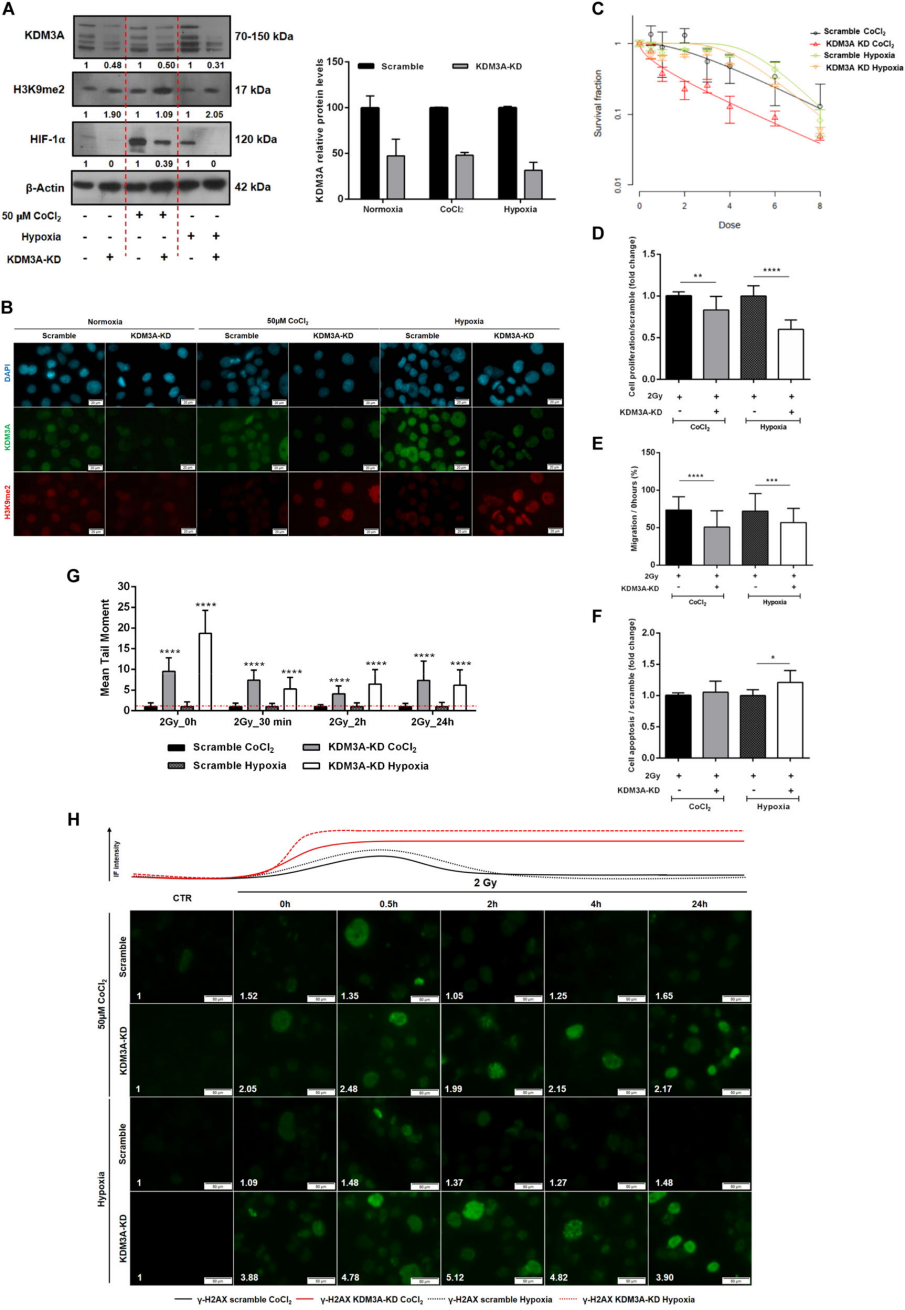
Radiosensitizing effect of KDM3A knockdown in Kyse-410 ESCC cell line. **a** Representative images of total protein levels of KDM3A (70–150 kDa), H3K9me2 (17 kDa), and HIF-1α (120 kDa) in KDM3A knockdown compared with scramble under normoxia and hypoxic conditions (50 µM CoCl_2_ or 0–1% O_2_). β-Actin (42 kDa) was used as loading control. **b** Representative IF pictures of co-localized nuclear DAPI (blue), KDM3A (green), and H3K9me2 (red) in KDM3A-KD and scramble, under normoxia and hypoxic conditions (50 µM CoCl_2_ or 0–1% O_2_). All pictures were taken with Olympus IX51 microscope at ×200 magnification (scale bar 50 μm). **c** Cell survival curves from Kyse-410 KDM3A-KD/Scramble cell lines irradiated with [0–8] Gy range IR dose fraction under 50 µM CoCl_2_ and 0.5–1% O_2_ hypoxic conditions through SHMT model analysis. Results are presented as mean ± SD of at least three independent experiments. **d** Cell proliferation (**e**) 24 h migration normalized to 0H and (**f**) cell apoptosis for combined KDM3A-KD/Scramble + 2 Gy irradiation under hypoxic conditions (50 µM CoCl_2_ or 0–1% O_2_). Fold changes were obtained after 2 Gy/0 Gy normalization. Results are represented as mean ± SD of at least three independent experiments; *p < 0.05; **p < 0.01; ***p < 0.001; ****p < 0.0001. **g** DNA damage of 2 Gy irradiated Kyse-410 KDM3A-KD/Scramble cells between 0 and 24 h 50 µM CoCl_2_ and hypoxia (0.5–1% O_2_), by comet assay. Results refer to the mean values of at least 50 comets per condition. All values of DNA fragmentation (tail moment) were normalized to control (0 Gy). Fold change of relative values KDM3A-KD were compared to control scramble. **h** Representative pictures of nuclear γ-H2AX staining of 2 Gy irradiated Kyse-410 KDM3A-KD/Scramble cells (0–24 h) under 50 µM CoCl_2_ and hypoxia (0.5–1% O_2_). All pictures were taken with Olympus IX51 microscope at ×200 magnification (scale bar 50 μm). IF quantification was done using ImageJ software (version 1.6.1, from National Institutes of Health) and represented as a fold change between 2 Gy irradiated cells and non-irradiated control. IF, fluorescence intensity.

To unveil whether KDM3A is implicated in ESCC radioresponse, *KDM3A* knockdown (KDM3A-KD) was performed in the Kyse-410 cell line (Fig. 4). KDM3A and HIF-1α protein expression decreased in KDM3A-KD- compared to scramble- Kyse-410 cells, both at baseline levels and with 50 µM CoCl_2_ or at 0.5–1% O_2_ (Fig. 4a). Conversely, increased expression of nuclear KDM3A-targeted H3K9me2 was depicted in KDM3A-KD cells (Fig. 4a, b). As expected, cell survival fraction decreased within CoCl_2_ and hypoxic conditions in KDM3A-KD cells, simultaneously with lower D_0_, D_q_, and SF2 values (Fig. 4c and Supplementary Table S2). Additionally, SER values higher than 1 were found in both hypoxic (50 µM CoCl_2_ and 0.5–1% O_2_) KDM3A-KD. Similarly, reduced proliferation and cell migration and higher apoptosis rates were showed by KDM3A-KD cells under hypoxic (50 µM CoCl_2_ and 0.5–1% O_2_) conditions after 2 Gy IR (Fig. 4d–f), suggesting cell aggressiveness impairment. Of note, global DNA damage intensity demonstrated by comet assay (Fig. 4g and Supplementary Fig. S2C) and γ-H2AX foci staining (Fig. 4h) remains overtime in KDM3A-KD-hypoxic irradiated cells and largely diverged from scramble-hypoxic status. Together, these results indicate that hypoxic-induced JmjC-KDMs modulation promotes ESCC cells’ radiosensitization, supporting KDM3A’s as a key RT responsiveness mediator.

### KDMs activity inhibition impairs DSB DNA damage repair after ionizing radiation exposure

**Fig. 5:**
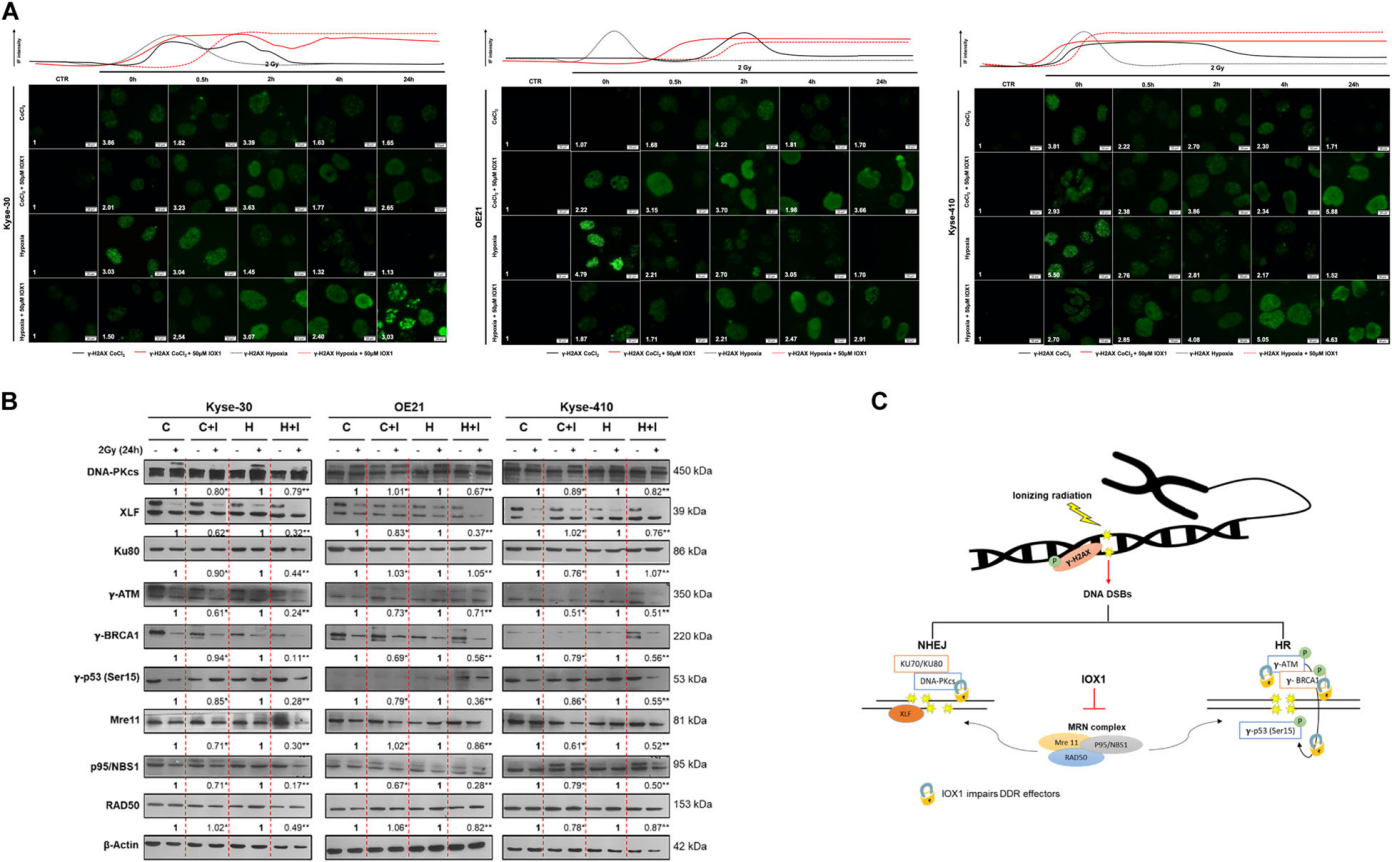
Effect of IOX1 inhibitor in DNA damage repair after 2 Gy irradiation in ESCC cell lines. **a** Representative pictures of nuclear γ-H2AX staining of 2 Gy irradiated ESCC cells treated with 50 µM IOX1 (0–24 h) under 50 µM CoCl_2_ and hypoxia (0.5–1% O_2_) conditions. All pictures were taken from Olympus IX51 microscope at ×400 magnification (scale bar 20 μm). ImageJ software (version 1.6.1, from National Institutes of Health) was used for IF quantification and represents a fold change between 2 Gy irradiated cells and non-irradiated control. IF, fluorescence intensity. **b** Representative images of total XLF (39 kDa), DNA-PKcs (450 kDa), Ku80 (86 kDa), γ-ATM (350 kDa), γ-BRCA1 (220 kDa), γ-p53 (53 kDa), Mre11 (81 kDa), NBS1(95 kDa), RAD50 (153 kDa) protein levels in ESCC cells treated with 50 µM IOX1 under 50 µM CoCl_2_ and hypoxia (0.5-1% O_2_) conditions before and after 24 h of 2 Gy irradiation. Fold change values of 2 Gy/0 Gy IOX1 were compared with 2 Gy/0 Gy hypoxic conditions alone. β-Actin (42 kDa) was used as loading control; C - 50 µM CoCl_2_; C + I - 50 µM CoCl_2_ + 50 µM IOX1; H – hypoxia; H + I – hypoxia + 50 µM CoCl_2_. **c** Schematic representation of DNA damage repair network. DSB, double-strand breaks; DDR, DNA damage repair; HR, homologous recombination; NHEJ, non-homologous end-joining.

To further understand whether KDMs activity inhibition with IOX1 treatment affects DNA damage repair (DDR), enriched γ-H2AX foci were found after 0.5 h 2 Gy IR until 24 h in all ESCC cells treated with IOX1, under hypoxic conditions (50 µM CoCl_2_ and 0.5–1% O_2_), indicating increased DSBs and less DDR (Fig. 5a). Accordingly, reduced DDR effectors relative protein levels were observed in hypoxic-IOX1 ESCC treated cells after 2 Gy IR (Fig. 5b, c). Of note, both homologous recombination (HR) and non-homologous end-joining (NHEJ) repair pathways were disturbed (Fig. 5c). Phospho-ATM (γ-ATM) and DNA-PKcs are common DNA damage kinases activated in response to DSBs formation in each repair pathway, HR and NHEJ, respectively^32^. Herein we found reduced γ-ATM and DNA-PKcs protein levels after 24 h of 2 Gy IR exposure in hypoxic-IOX1 treated ESCC cells compared with respective hypoxic controls, suggesting a DDR network deficiency (Fig. 5b, c). Although DNA-PKcs activation was maintained after 24 h of 2 Gy IR it was less pronounced in IOX1 treated cells (Fig. 5b, c). Because DNA-PKcs is known to critically interact with Ku70/80 heterodimer to signalize DDR kinase activity during classic NHEJ repair pathway we tested that in our cells^33^. Overall, hypoxic-IOX1 treated cells displayed less DNA-PKcs activation, although subtle differences were found for Ku80 protein expression (except for Kyse-30 cells with 0.5–1% O2 + 50 µM IOX1), suggesting that IOX1 did not consistently influence Ku80 expression (Fig. 5b). Conversely, NHEJ factor 1 or Cernunnos, also known as XRCC4-like factor (XLF), which is another critical core component of NHEJ repair pathway to endure gap-filling^34^, exhibited reduced activation in hypoxic-IOX1 treated cells compared with respective hypoxic controls, after 24 h of 2 Gy IR exposure, with the exception for kyse-410 with 50 µM (CoCl_2_ + IOX1) (Fig. 5b, c).

Moreover, IOX1 reduced the relative protein expression of all studied HR components (Fig. 5b, c), in accordance with NHEJ network variations. Additionally, defects on the major DDR mechanisms were more evident with IOX1 addition in hypoxic-ESCC cells (Fig. 5b, c).

Finally, IOX1 strongly reduced protein levels of both Mre11 and p95/NBS1 after 24 h of IR, suggesting a compromised DDR complex activity and further supporting previous results (Fig. 5b, c). Taken together, these findings reveal that DDR proteins hypoxic-dependent modulation after IR exposure was mainly abrogated with JmjC-KDMs activity inhibition by IOX1, in all ESCC cell lines.

### KDM activity inhibition decreases in vivo tumor growth and proliferation

**Fig. 6:**
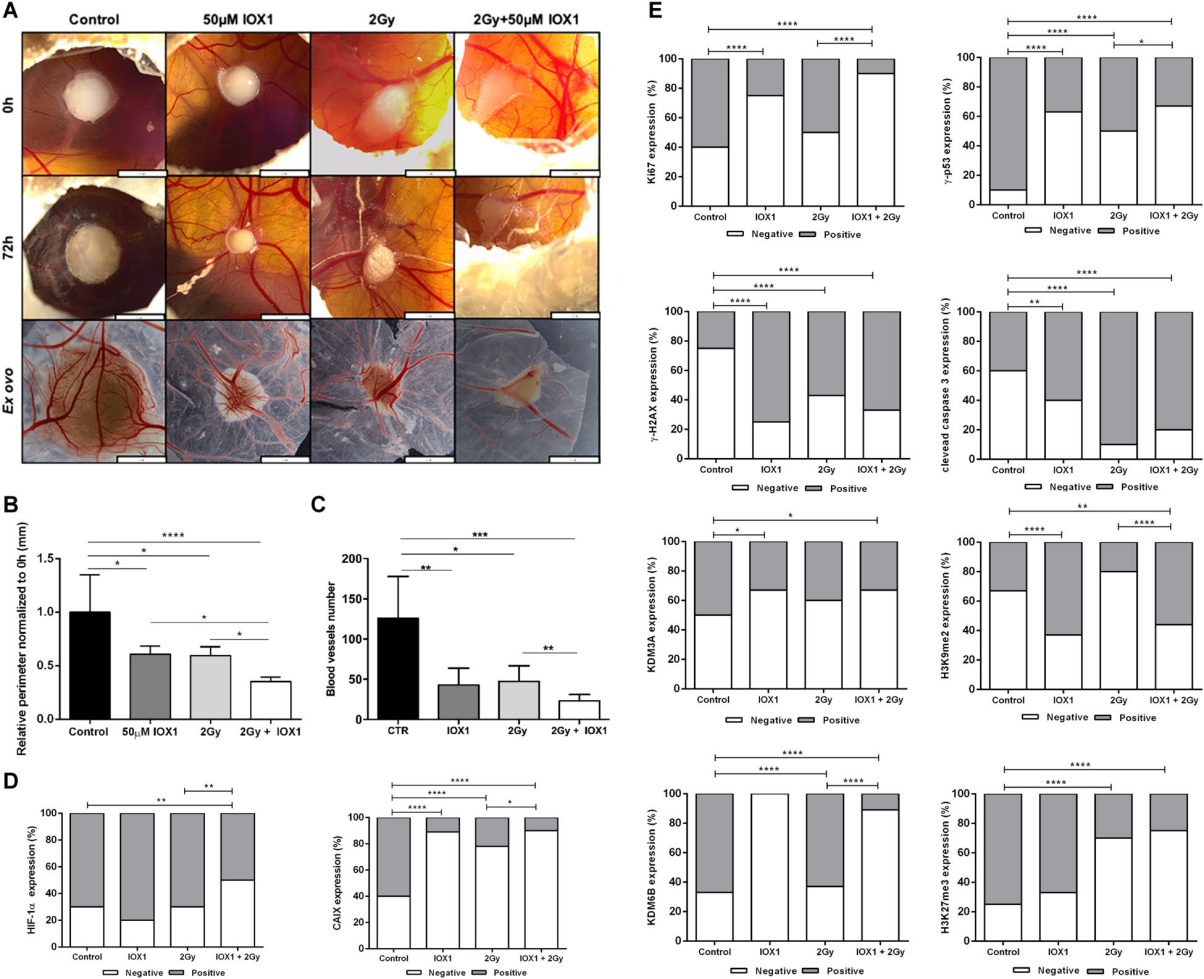
IOX1 effect in irradiated ESCC microtumors in in vivo CAM assay. **a** Representative pictures of CAM microtumors for treatment conditions at 0 h and 72 h. Digital images were taken under a stereomicroscope Olympus S2X16 using a digital camera Olympus SC180 (scale bar 1 mm). Relative perimeter (mm) (**b**) and blood vessel recruitment (**c**) of ESCC microtumors normalized to 0 h of drug treatment. Results are presented as mean±SD of 10 eggs per group condition; *p < 0.05; **p < 0.01; ***p < 0.001. Graphical re*p*resentation of (**d**) hypoxic markers (CAIX and HIF-1α) and (**e**) Ki-67, γ-p53, cleaved caspase 3 and γ-H2AX, as well as KDM3A, KDM6B, H3K9me2, and H3K27me3 immunostaining for ESCC microtumors sections; *p < 0.05; **p < 0.01; ***p < 0.001; ****p < 0.0001.

Overall, IOX1 combined with irradiation disclosed a more impressive effect in ESCC microtumor perimeter after 72 h. Nevertheless, a significant reduction in chorioallantoic membrane (CAM) microtumors size was also achieved with each treatment alone (Fig. 6a, b). These results were paralleled by the reduced number of blood vessels formation (Fig. 6c). Furthermore, the combined treatment led to a significant decrease in CAIX and HIF-1α expression (Fig. 6d and Supplementary Fig. S4).

Additionally, an overall decrease of Ki-67 and γ-p53 expression was displayed by CAM microtumors treated with IOX1 alone or combined with radiation, compared with the respective control (Fig. 6e and Supplementary Fig. S4). Conversely, increased cleaved caspase 3 and γ-H2AX expression was depicted by treated CAM microtumors, although no differences were apparent between tumors exposed to combined treatment or irradiated only (Fig. 6e and Supplementary Fig. S4).

Finally, KDM3A and KDM6B decreased expression was accomplished in CAM tumors treated with IOX1 inhibitor (Fig. 6e and Supplementary Fig. S4), although only H3K9me2 was significantly increased in target marks (Fig. 6e and Supplementary Fig. S4), supporting in vitro findings, and thus, indicating KDM3A as a key targetable molecule to radiosensitize hypoxic ESCC.

### KDM3A is overexpressed in ESCC tissues

**Fig. 7:**
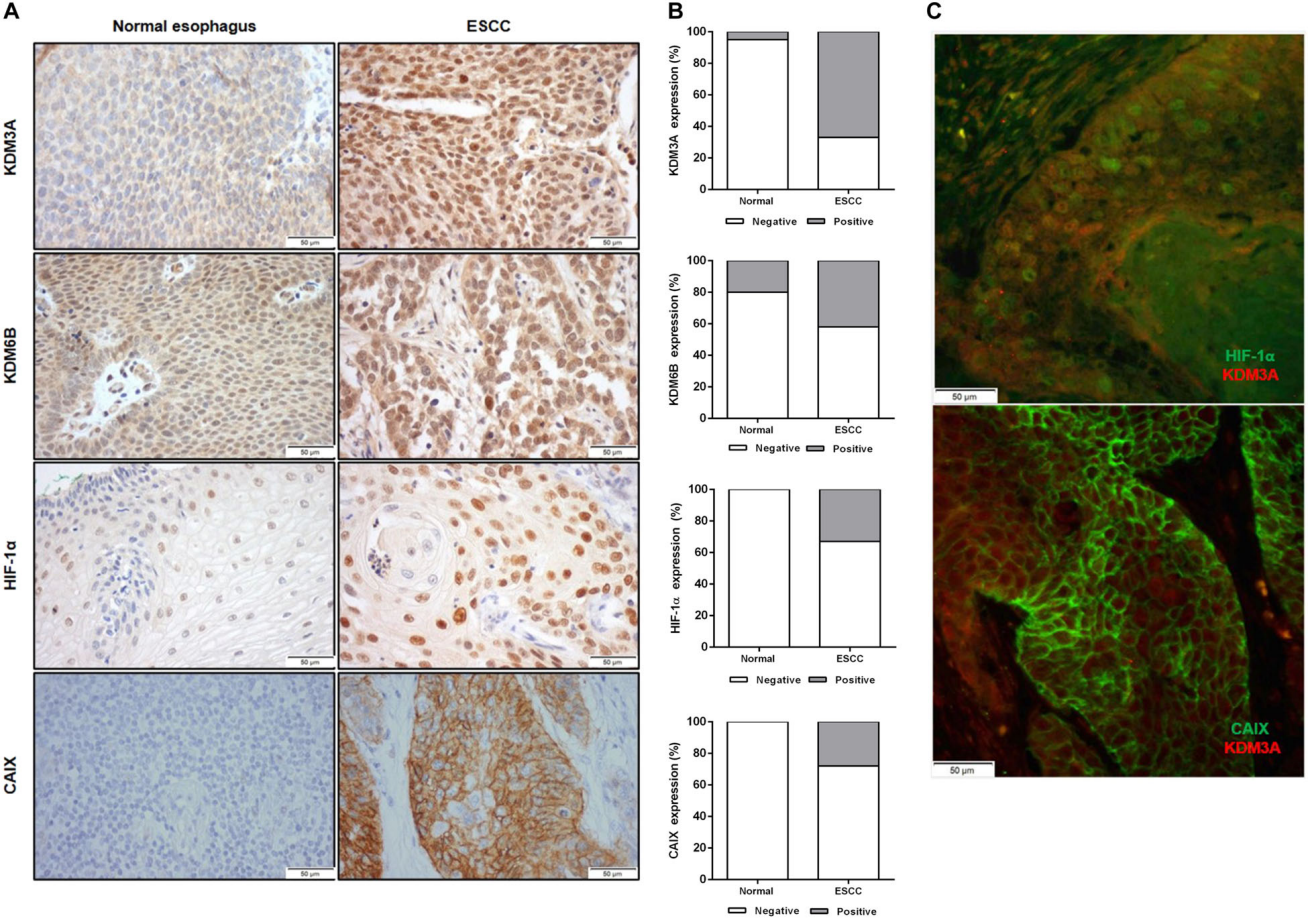
JmjC-KDMs expression in ESCC and normal esophagus tissue samples. **a** Representative IHC images for KDM3A, KDM6B, HIF-1α, and CAIX expression in NE and ESCC tissues. Pictures were taken from a microscope Olympus BX41 with a digital camera Olympus U-TV0.63XC, at ×200 magnification (scale bar 50 μm). **b** Graphical representation of % protein expression in ESCC and NE. **c** IF co-localization of KDM3A (red fluorescence) and HIF-1α and CAIX (green fluorescence) in ESCC tissue. Representative pictures were taken from Olympus IX51 microscope at ×200 magnification (scale bar 50 μm).

In tissue samples, nuclear KDM3A protein expression was significantly higher in ESCC than in normal esophagus (NE) (67% vs 5%, *p* < 0.0001), whereas no differences were apparent concerning KDM6B expression (42% vs 20%, *p* = 0.019) (Fig. 7a, b and Table 1). Moreover, specific nuclear and cell membrane expression was observed for HIF-1α and CAIX, respectively. Both HIF-1α (33% vs 0%, *p* = 0.002) and CAIX (28% vs 0%, *p* = 0.009) expression were upregulated in tumors compared to NE (Fig. 7a, b and Table 1).

**Table 1:** KDMs and hypoxic markers positive expression in ESCC and normal esophagus.

	*N*	KDM3A (%)	KDM6B (%)	HIF-1α (%)	CAIX (%)
Normal	20	1 (5)	4 (20)	0 (0)	0 (0)
ESCC	61	41 (67)	25 (42)	20 (33)	17 (28)
	*p value*	*<0.0001*	*0.109*	*0.002*	*0.009*

Interestingly, a significant association between KDM3A and HIF-1α (*p* = 0.021) expression was found, although only a trend was observed regarding CAIX (*p* = 0.07) (Table 2). Indeed, in ESCC tissues, KDM3A co-localized both with HIF-1α and CAIX (Fig. 7c). Nonetheless, no association was found for KDM6B and any of hypoxic markers (Table 2).

**Table 2:** KDMs and hypoxic markers association in tissue cohort.

	KDM3A (%)		*p value*	KDM6B (%)		*p value*
	Negative	Positive		Negative	Positive	
HIF-1α (%)						
Negative	34 (42)	27 (33)	*0.021*	41(51)	20 (25)	*0.282*
Positive	5 (6)	15 (19)		10 (13)	9 (11)	
CAIX (%)						
Negative	34 (42)	30 (37)	*0.07*	40 (50)	23 (29)	*1*
Positive	5 (6)	12 (15)		11 (14)	6 (7)	

## Discussion

ESCC is highly incident worldwide, entailing poor prognosis and low overall survival rates^1,35^, despite some therapeutic advances over the last years^7,36^. Thus, the identification of new molecular targets that might improve therapeutic efficacy for advanced ESCC is urgently needed. Hypoxia has been associated with poor prognosis in EC, namely due to resistance to RT, the gold standard therapy for advanced stages^12,37^. Indeed, EC patients with non-hypoxic tumors, displaying HIF-1α downregulation, endure complete chemoradiotherapy response, contrary to patients with high HIF-1α expressing tumors^14^. Herein, we proved that in vitro ESCC cells under hypoxia demonstrate a decreased RT response, through a HIF-1α-dependent manner. Furthermore, reduced γ-H2AX expression, lower DNA fragmentation and decreased cell death fraction was found in tumor cells under hypoxia after IR exposure.

Recently, hypoxia has been associated with epigenetic deregulation^21^. Indeed, histone lysine demethylases, namely JmjC-KDMs superfamily was found to be regulated by oxygen levels and/or HIF-1α transcription factor^38^. Specifically, both low O_2_ levels and HIF-1α expression were reported to induce KDM3A and KDM6B expression^23^. Still, HIFs might be regulated by 2-oxoglutarate-dependent members, which involves JmjC-KDMs family, as well as, prolyl hydroxylases (PHD)^23^. Accordingly, in our hands reduced nuclear HIF-1α expression levels were found in KDM3A-KD cells.

Furthermore, nuclear HIF-1α expression levels were not significantly altered in IOX1 CAM-associated microtumors, whereas a significant reduction was found after 2 Gy IR and after combined treatment. This was followed by decreased tumor volume and consequently, decreased hypoxic foci. Additionally, IOX1 was also shown to have a higher selectivity for Pan-JmjC-KDMs activity inhibition than PHDs^39^. Indeed, a similar HIF-1α expression trend in IOX1 Kyse-410 microtumors may be partially explained by intrinsic expression levels in the control condition and the apparent hypoxic foci in 3D tumors.

Additionally, findings from in vitro assays using different cell lines suggested that under hypoxic conditions, HIF-1α recruits KDM3A, promoting H3K9me2 demethylation, and increasing gene transcription^40^. Remarkably, we showed that in ESCC cells, both KDM3A and KDM6B expression was upregulated in parallel with low oxygen levels and /or HIF-1α overexpression, with the latter specifically bound to KDM3A and KDM6B promoter region under hypoxic conditions. Thus, our findings both confirm and extend previously published observations.

Additionally, several studies suggested that both KDM3A and KDM6B were putative therapeutic targets in different cancer models, not including ESCC. Indeed, KDM3A inhibition decreased estrogen receptor positive breast cancer cells’ proliferation^41^ whereas it was implicated in stemness and chemoresistance in ovarian cancer^42^. Furthermore, KDM3A targeting increased response to anti-angiogenic therapies, disclosing a role in tumor angiogenesis^43^ Interestingly, in vitro studies in lung and breast cancer cells demonstrated that KDM6A inhibition decreased cell survival and improved RT response, through H3K27me3 enhancement^44^. Furthermore, two other KDMs, KDM4C, and PHF8 were associated with ESCC malignant features. Indeed, KDM4C targeting decreased ESCC stemness properties^45^, whereas PHF8’s inhibition promoted apoptosis and decreased ESCC cell proliferation and invasion^46^. Interestingly, our in vitro data we demonstrated that radioresistant phenotype observed under hypoxic conditions was abrogated with both JmjC-KDMs activity inhibition and KDM3A-KD, promoting radiosensitization in ESCC cells, in line with the results obtained with IOX1 inhibitor. Of note, hypoxic-dependent KDM3A seems to play a critical role in RT response modulation in in vitro and in vivo experiments. Furthermore, radiosensitized hypoxic-IOX1 treated ESCC cells impaired DDR network, with decreased relative protein levels of the major DDR effectors. In the same vein and as a consequence of DNA damage repair deficiency, γ-H2AX was independently maintained overtime after cell replication in hypoxic-induced IOX1 cells, suggesting the persistence unrepaired DNA DSBs. Remarkably, a similar function of a KDM5B inhibitor, JIB-04, was reported to radiosensitize lung cancer^47^. Defects in DNA repair dynamics prevents DDR resolution, due to endless γ-H2AX activation and impaired recruitment of the major HR and NHEJ repair effectors^47^.

Those results were further supported by in vivo experiments using the CAM assay. Additionally, the reduction on microtumor aggressiveness features was demonstrated by reduced Ki67 expression and γ-p53 expression, thus supporting the DDR-related effectors decreased expression, while DNA DSB levels were increased, as well as cell death, with active caspase 3 and γ-H2AX overexpression. Importantly, both in vitro and in vivo experiments support the demethylating role of KDM3A as a mediator of radioresistance, since higher H3K9me2 levels were observed in IOX1 treated tumors. Interestingly, deregulation of JmjC-KDMs, including LSD1^48^, KDM3C^49^, KDM4C^50^, KDM7B^46^, and UTX/KDM6A^51^, has been implicated in esophageal carcinogenesis, although published data concerning KDM3A and KDM6B is notoriously lacking. Indeed, our study is the first to demonstrate that, in primary ESCC, KDM3A overexpression positively associates with the hypoxic markers, HIF-1α and CAIX, further supporting the in vitro data^40^.

Overall, our results indicate that hypoxia induces KDM3A overexpression, conferring growth advantage to ESCC submitted to IR, highlighting its role in cancer progression and therapy resistance. Remarkably, in ESCC, KDM3A might constitute a promising therapeutic target for pharmacological inhibition in combination with neoadjuvant RT to improve ESCC patients’ survival.

## Materials and methods

### ESCC cell lines and cell culture

For this study, three ESCC cell lines obtained from American Type Culture Collection (ATCC®), USA, were used for in vitro and in vivo assays: Kyse-30 (well differentiated), OE21 (moderately differentiated), and Kyse-410 (poorly differentiated). Additionally, for drug toxicity evaluation, a normal epithelial cell line from human esophagus, Het-1A (ATCC®) was used. Tumor cells were grown in RPMI-1640 medium (Biochrom, Merk, Germany), while normal cells were maintained in Dulbecco’s Modified Eagle’s Medium (DMEM 1x, GBICO, Invitrogen, USA), supplemented with 10% fetal bovine serum (FBS, Biochrom, Merk, Germany) and 1% penicillin/streptomycin (GIBCO, Invitrogen, USA) at 37 °C with 5% CO_2_ and 74% N_2_. Mycoplasma test was performed using TaKaRa PCR Mycoplasma Detection Set (Clontech Laboratories, EUA), before all experiments.

### Hypoxia stabilization

Hypoxia induction was carried out for in vitro assays using airtight chambers [modular incubator chamber (MIC-101), Billups-Rothenberg, USA] saturated with 95% N_2_ and 5% CO_2_. ESCC cell lines were grown at 0.5–1% O_2_, in an incubator at 37 °C.

### Chemicals

Cobalt Chloride, CoCl_2_ (Sigma-Aldrich, Germany) was dissolved in sterile distilled water (dH_2_O) at 50 μM^52^. Furthermore, 5-Carboxy-8-hydroxyquinoline (IOX1, Sigma-Aldrich, Germany), the most potent pan-histone demethylase inhibitor^39^, was used. This compound was dissolved in dimethyl sulfoxide (DMSO, Sigma-Aldrich, Germany) at 20 and 50 μM depending on the assay.

### Irradiation

IR was performed at room temperature (R/T) with normal oxygen levels, using TrueBeam linear accelerator as irradiation source within a field of 25×25 cm^2^, a photon energy of 6MV and a dose rate of 600 MU/min. For hypoxic experiments (0.5-1% O_2_) cells were maintained under oxygen deprivation during all experimental timeline, before and after IR radiation exposure for all in vitro assays. Nonetheless, In vivo chicken CAM irradiation was carried out in a microSelectronv3 Iridio-192 brachytherapy (192-Ir-mHDR-v2r) at 2 Gy per egg/pulse for chicken embryo’s protection (Image planning represented on Supplementary Fig. S5) since only a low dose rate brachytherapy was attained by the animal.

### RNA extraction, quantification, cDNA synthesis and RT-qPCR

Tumor cell RNA was extracted by a ribozol reagent method. Revert Aid RT Kit (ThermoScientific Inc.) was used for cDNA synthesis, according to the manufacturer’s instructions. RT-qPCR was performed in LightCycler480II (Roche) using Xpert Fast SYBER Mastermix Blue (GE22.2501, Grisp) with specific designed primers (Supplementary Table S3). GUSβ was used as endogenous control.

### Total protein extraction, quantification, and SDS-PAGE western blot

For next experiments all antibodies details are described in Supplementary Table S4.

Briefly, cells were scraped in lysis Buffer (Kinexus Bioinformatics Corporation, Vancouver, British Columbia, Canada) on ice. Protein quantification was performed using the Pierce BCA Protein Kit (Thermo Scientific Inc.), according to the manufacturer’s instructions. Western blot (WB) was performed as previously described^53^ using specific primary antibodies. After primary antibody incubation overnight at 4 °C, specific conjugated horseradish peroxidase secondary antibodies (Bio-Rad, USA) were incubated 1 h R/T. Chemiluminescence was detected with Clarity WB ECL substrate (Bio-Rad, USA) and evaluated using ImageJ software (version 1.6.1, from National Institutes of Health). β-actin served as control of the total loaded protein.

### Immunofluorescence and immunocytochemistry

ESCC cells were seeded in cover slips into culture plates and fixed with 4% paraformaldehyde. For nuclear proteins, cells were permeabilized with 0.25% Triton X-100 solution in 1x phosphate-buffer saline (PBS 1x).

For IF, cells were blocked with 5% bovine serum albumin (BSA) (Santacruz Biotechnology, Inc, USA) in PBS 1x. Except for γ-H2AX antibody, which was incubated for 1 h at R/T, all remainder antibodies (H3K9me2, H3K27me3, KDM3A, and KDM6B) were incubated overnight at R/T. Then, cells were incubated for 1 h at R/T with secondary antibodies, anti-rabbit immunoglobulin G (IgG) Alexa Fluor^TM^ 488 goat (A11008, Invitrogen, Thermofisher Scientific, USA) and/or anti-mouse IgG Alexa Fluor^TM^ 594 goat (A11032, Invitrogen, Thermofisher Scientific, USA) and stained with 4’6-diamidino-2-phenylindole (DAPI) (AR1176, BOSTER Biological Technologies, China).

For immunocytochemistry (ICC), NovolinkTMMax Polymer Detection System (Leica Biosystems, Cat. #RE7260-K) was used. Briefly, endogenous peroxidases activity was blocked with 3% H_2_O_2_, followed by non-specific linked blockage in horse serum (GIBCO, Invitrogen, USA). Primary antibody was incubated overnight at R/T in a humidified chamber. Furthermore, cells were incubated with post-primary solution followed by polymer and 3,3’-diaminobenzidine (DAB) (Sigma-Aldrich^TM^, Germany), Then, cells were counterstained with hematoxylin and mounted in aqueous medium (Aquatex®, Merk, Germany).

### *KDM3A* gene knockdown

*KDM3A* gene knockdown (KDM3A-KD) was performed using CRISPR-cas9 technology with a guide RNA (gRNA) sequence targeting *KDM3A* (GenScript, Piscataway, NJ) (Supplementary Table S3). Briefly, plasmid transfection was carried out with Lipofectamine^®^ 3000 reagent (Invitrogen, USA), following manufacturing instructions. Scramble gRNA sequence was used as negative transfection control. Transfected cells’ selection was done by Puromycin at 1 μg/mL in RPMI-1640 cell culture medium.

### Phenotypic assays

IOX1 effects on cell viability were assessed by MTT assay, following previously reported procedures^54^. Apoptosis was evaluated after 24 h of 2 Gy IR and after 48 h and 72 h of IOX1 induction and hypoxic stimulation, respectively, using APOPercentage assay kit (Biocolor Ltd., Belfast, Northern Ireland, UK), according to manufactured instructions. Concerning wound-healing assay, wild-type (WT) ESCC cells or Kyse-410 KDM3A-KD and scramble were seeded and exposed to 50 µM CoCl_2_ or hypoxia. Subsequently, when applicable, cells were treated with 50 µM IOX1 24 h before 2 Gy IR. Then, cells growth at 95% of confluence and two parallel “wounds” in each well (initial slope) were done. Then, relative migration distance was analyzed by beWound - Cell Migration Tool (version 1.5) calculating % cell migration = (A/B)/C*100 (A, width of cell wound at initial slope; B, width of cell wound at several time points; C, width mean of cell wound at initial slope)]. KDM3A-KD cell proliferation assay was assessed after 24 h of 2 Gy IR and 48 h of 50 µM CoCl_2_ and hypoxia induction, using Cell proliferation ELISA BrdU (5-bromo-2’deoxyuridine) assay kit (Roche Applied Sciences, Penzberg, Germany), according to manufactured instructions.

### Colony formation

ESCC cells were seeded in 6-well culture plates at specific concentrations for each experimental group, as detailed in Supplementary Table S1. Then, after 48 h of hypoxia exposure or CoCl_2_ addition, cells were exposed to IR and incubated at 37 °C for 7 days. Experiments were carried out in all ESCC cell lines, whereas Kyse-30 cell line was not able to form colonies after CoCl_2_ chemical induction. Also, 24 h after hypoxia stimulation and before IR, cells were treated with 20 µM IOX1. Colonies were stained with 25% (w/v) Giemsa. Colonies depicting more than 50 single cells were counted and analyzed using RAD ADAPT software (Biomedical Simulation Resource, USC, California, USA)^55^. Exponential single hit multi-target model (SHMT), S(D) = PE * [1-(1-exp (-D/D_0_))^n^) was used. Concerning statistics, D_0_ represents the induction of one lethal event per cell becoming at 37% of viability, through the measurement of the ending slope resulting from a multiple event killing. Furthermore, D_q_, quasi-threshold dose represents the width of the curve shoulder. Additionally, sensitized enhancement ratio (SER) was evaluated according to D_0_ (without sensitizer) / D_0_ (with sensitizer). The sensitizer is IOX1 inhibitor or Kyse-410 KDM3A-KD cells.

### Alkaline comet assay

ESCC cells were treated with 2 Gy IR at 0 h, 0.5 h, 2 h, and 24 h. Briefly, cells were re-suspended in 0.5% low melting agarose (w/v) and immediately placed on a sheet previously covered with 1% normal melting agarose (w/v). Then, a cell lysis buffer (2.5 M NaCl, 100 mM Na_2_EDTA, 10 mM Tris Base, 1% Triton X-100), pH 10 was added. Electrophoresis was performed for 30 min at 21 V, 300 mA, 4 °C. Lastly, the sheets were incubated in a neutralization buffer (0.4 M Tris-Base, pH 7.5,), followed staining with SybrGreen. Comet analysis was done using OpenComet v.1.3.1^56^. Global DNA damage (SSB and DSB)^57^ evaluation was determined by measuring tail moment (tail % DNA x means of head x tail distance) and representative pictures taken with Olympus IX51 microscope at ×200 magnification. A sampling of at least 50 comets was included in the analysis.

### ChIP – qPCR

Firstly, DNA crosslink was done using 1% formaldehyde at R/T in 1 × 10^7^cells. Next, at 4 °C, cell lysis buffer was added (10 mM Tris-HCl, 10 mM NaCl and 0.5% NP-40), followed by a nuclei lysis buffer (50 mM Tris-HCl, 10 mM EDTA and 1% SDS). Then, cells were sonicated within optimized cycles. DNA fragments must be ideally with 300 to 500 base pairs (bp). Protein/DNA binding, using a primary antibody, sonicated chromatin, and Magn ChIP protein A + G beads (EMD Millipore, USA), was done. Then, consecutive fully washes were carried out using low salt buffer (0.1% SDS, 1% Triton X-100, 2 mM EDTA, 20 mM Tris-HCl, pH = 8.1, 150 mM NaCl), high salt buffer (0.1% SDS, 1% Triton X-100, 2 mM EDTA, 20 mM Tris-HCl, pH=8.1, 500 mM NaCl) and LiCl buffer (0.25 M LiCl, 1% NP-40, 1 % Desoxicolate, 1 mM and EDTA, 10 mM Tris). Finally, DNA purification was carried out using a Qiaquick gel extraction kit (Qiagen, Germany) according to the manufacturer’s instructions, followed by RT-qPCR. Data is presented using %Input [100*(2^ (C_T_ Raw mean – C_T_)]. Normal mouse IgG and RNA polymerase II protein immunoprecipitation were used as internal controls.

### Tissue immunoexpression

Formalin-fixed paraffin embedded (FFPE) primary tumors from ESCC patients (n = 61) were selected from Portuguese Oncology Institute of Porto (IPO-Porto), Portugal after informed patient consent and inserted into tissue microarrays (TMAs). Three representative cores were included for each case. Furthermore, NE samples (n = 20), were obtained from the esophageal margin of radical gastrectomies. This study was approved by the Institutional Review Board of IPO-Porto (CES IPO: 202/017).

Immunohistochemistry (IHC) was performed for CAIX, HIF-1α, KDM3A, and KDM6B, as previously described^58^. Semi-quantitative analysis was blindly performed by an expert pathologist classifying extension (0: <10%; 1: 10-50%; 2: 50-75%; 3: >75%) and intensity (0: negative; 1: weak; 2: moderate; 3: strong) for each protein. Then, the combination of intensity and extension was done and a final score ≥1 was set to define positive cases.

### CAM assay

Fresh fertilized eggs (PintoBar, Lda, Portugal) were incubated at 37 °C in a humid environment. After 6 days of embryonic development, a window was opened into the eggshell under aseptic conditions. On day 10, Kyse-410 cells suspension in growth factor-reduced Matrigel (BD Biosciences) were seeded on CAM. Then, on day 13, a treated group, randomly selected, received IOX1 50 μM whereas a control group received only 1% DMSO in complete RPMI-1640. After 24 h, CAM was irradiated with 2 Gy. Lastly, on day 17, tumors were dissected and included in a paraffin block. Microtumor images were obtained on day 13 (0 h of treatments) and at day 17 (72 h of treatment). Relative perimeter in *in ovo* was assessed using CellSens software (version V0116, Olympus). *Ex ovo* pictures were obtained for blood vessels counting using Image J software.

Immunostaining of microtumors’ sections was evaluated through a quantitative method using GenASIS software (Applied Spectral Imaging, ASI). Staining’s evaluation was performed as described in the tissue immunoexpression subsection.

### Statistics

Non-parametric tests (Kruskal–Wallis or Mann–Whitney U test) among groups with Bonferroni’s correction were used to compare different conditions in in vitro assays through GraphPad Prism version 6.0. IHC results were analyzed by Pearson’s chi-square or Fisher’s exact test, using the SPSS 25.0 software. All results are shown as the mean ± SD for each group. For each analysis, p values were considered significant when inferior to 0.05 (*p < 0.05; **p < 0.01; ***p < 0.001; ****p < 0.0001).

## Supplementary information

Supplementary Tables

Supplementary figure S1

Supplementary figure S2

Supplementary figure S3

Supplementary figure S4

Supplementary figure S5

Supplementary Figure Legends
