# Supplementary material for: JmjC-KDMs KDM3A and KDM6B modulate radioresistance under hypoxic conditions in esophageal squamous cell carcinoma
Source: Cell Death Dis. 2020 Dec 14;11(12):1068. doi: 10.1038/s41419-020-03279-y (PMC7736883; doi:10.1038/s41419-020-03279-y)
Supplement: Supplementary file 1 — Supplementary Tables [file 41419_2020_3279_MOESM1_ESM.docx]

**Macedo-Silva et al_Supplementary tables**

**Supplementary table S1.** Statistical parameters of single-hit multi target model for irradiation analysis

|  |  | D0_0_ (Gy) | Dq_q_ (Gy) | SF2 | Cells Seeded | | *p value* | |
| --- | --- | --- | --- | --- | --- | --- | --- | --- |
| Kyse-30 | **Normoxia** | 1.73 | 0.84 | 0.30 | 1000 | - | |  |
|  | **Hypoxia** | 2.92 | 2.73 | 0.33 | 1000 | *0.002* | |  |
| OE21 | **Normoxia** | 1.26 | 1.85 | 0.39 | 1300 | *-* | |  |
|  | **CoCl_2_** | 1.30 | 2.12 | 0.50 | 2300 | *0.016* | |  |
|  | **Hypoxia** | 1.65 | 3.44 | 0.74 | 1300 | *0.032* | |  |
| Kyse-410 | **Normoxia** | 2.73 | 1.95 | 0.70 | 2500 | *-* | |  |
|  | **CoCl_2_** | 3.69 | 1.59 | 0.84 | 3000 | *0.730* | |  |
|  | **Hypoxia** | 2.25 | 2.64 | 0.64 | 2500 | *0.080* | |  |

**Supplementary table S2.** Statistical parameters of single-hit multi target model for IOX1/CRISPRcas9 + irradiation analysis

|  |  | D0_0_ (Gy) | Dq_q_ (Gy) | SF2 | SER | Cells Seeded | |
| --- | --- | --- | --- | --- | --- | --- | --- |
| Kyse-30 | **Hypoxia** | 2.92 | 2.29 | 0.77 | n.a | 1000 |  |
|  | **Hypoxia + IOX1** | 0.75 | 0.55 | 0.15 | 3.89 | 5000 |  |
| OE21 | **CoCl_2_** | 1.30 | 2.12 | 0.50 | n.a | 2300 |  |
|  | **CoCl_2_ + IOX1** | 0.98 | 1.34 | 0.34 | 1.33 | 5000 |  |
|  | **Hypoxia** | 1.65 | 3.44 | 0.92 | n.a | 1300 |  |
|  | **Hypoxia + IOX1** | 1.23 | 2.71 | 0.45 | 1.34 | 5000 |  |
| Kyse-410 | **CoCl_2_** | 3.69 | 1.59 | 0.70 | n.a | 3000 |  |
|  | **CoCl_2_ + IOX1** | 1.86 | 0.23 | 0.45 | 1.98 | 5000 |  |
|  | **Hypoxia** | 2.25 | 2.64 | 0.95 | n.a | 2500 |  |
|  | **Hypoxia + IOX1** | 1.41 | 1.85 | 0.63 | 1.60 | 5000 |  |
|  | **CoCl_2_ Scramble** | 2.75 | 2.272 | 1.30 | n.a | 3000 |  |
|  | **CoCl_2_ KDM3A-KD** | 2.048 | -2.413 | 0.22 | 1.34 | 3000 |  |
|  | **Hypoxia Scramble** | 1.85 | 3.651 | 0.81 | n.a | 2500 |  |
|  | **Hypoxia KDM3A-KD** | 1.57 | 2.77 | 0.67 | 1.18 | 2500 |  |

**Supplementary table S3.** RT-qPCR primers, ChiP qPCR primers and gRNA sequences details.

| **Technique** | **Gene** | **Primer designation** | **Distance from TSS (bp)** | **Forward sequence (5’-3’)** | **Reverse sequence (5’-3’)** | **T annealing (ºC)** |
| --- | --- | --- | --- | --- | --- | --- |
| RT-qPCR | Gusβ | - | - | CACTGAAGAGTACCAGAAAAGTC | TCTCTGCCGAGTGAAGATCC | 60ºC |
|  | KDM3A | - | - | GTGGGGATTGATTTGGACAC | TCTTCATCCGGTAGCAGTCC | 62ºC |
|  | KDM6B | - | - | GGGTGATGATTGGCTTTCTG | TGATAAGAGTGCCCGCTACC | 62ºC |
| ChIP - qPCR | KDM3A | A | 689 | TGGGAGGCATGAGTTCTTCT | TGTGTGCTCTGGACCTGAAG | 62ºC |
|  |  | B | 1242 | TCACCTTAGGATAGGCCATGTT | TGGTAGCCAAATGAAAGCAG | 60ºC |
|  | KDM6B | A | 732 | TAGGACTCAGGCTGGCTTGT | AAACCCGTGATCCCTCTTCT | 60ºC |
|  |  | B | 1115 | TCTTGGCCTTGGACTTATGC | TATCCTGTTTCCCCATCACC | 60ºC |
|  |  | C | 1367 | AGTGTCATCAGCACAGCCACAG | CACTTGGGGTCCCAATCTAA | 60ºC |
| CRISPR/cas9 | KDM3A gRNA | - | - | CGGAGAAAGTTGGCCGGTAT TGG | | - |
|  | Scramble gRNA | - | - | GCACTACCAGAGCTAACTCA | | - |

gRNA, guide RNA; TSS, transcriptional starting site.

**Supplementary table S4**. Antibody details for IHC, IF, ICC, WB and ChIP.

| **Primary antibody** | **Company** | **IHC** | | | **IF dilution** | **ICC dilution** | **WB dilution** | **ChIP dilution** |
| --- | --- | --- | --- | --- | --- | --- | --- | --- |
|  |  | ***Antibody dilution*** | ***Antigen retrieval buffer*** | ***Positive control*** |  |  |  |  |
| β-Actin | A1978, Sigma-Aldrich | - | - | - | - | - | 1:10000 1h; RT | - |
| γ-ATM | D25E5 (Ser1981), cell signaling | - | - | - | - | - | 1:1000 ON; 4ºC | - |
| γ-BRCA1 | 9009T (Ser1524), cell signaling | - | - | - | - | - | 1:1000 ON; 4ºC | - |
| γ-H2AX | 2577s, cell signaling | 1:150 ON; RT | Citrate 10mM, pH=6 | Invasive urothelial carcinoma | 1:150 ON; RT | - | - | - |
| γ-p53 | 9286T (Ser15), cell signaling | 1:200 ON; RT | Citrate 10mM, pH=6 | Ovarian cancer | - | - | 1:1000 ON; 4ºC | - |
| CAIX | 5649s, cell signaling | 1:100 ON; RT | Citrate 10mM, pH=6 | Clear cell renal cell carcinoma | - | 1:100 ON; RT | 1:1000 ON;  4ºC | - |
| Cleaved Caspase 3 | 9661s, cell signaling | 1:200 ON; RT | Citrate 10mM, pH=6 | Ovarian cancer | - | - | - | - |
| DNA-PKcs | 4602s, cell signaling | - | - | - | - | - | 1:1000 ON;4ºC | - |
| H3K27me3 | 07-449, Millipore | 1:250 ON; RT | Citrate 10mM, pH=6 | Ovarian cancer | 1:250 ON; RT | - | 1:250 ON;  4ºC | - |
| H3K9me2 | 4658s, cell signaling | 1:250 ON; RT | Citrate 10mM, pH=6 | Invasive urothelial carcinoma | 1:250 ON; RT | - | 1:250 ON; 4ºC | - |
| HIF-1α | 36169s, cell signaling | 1:500 ON; RT | 1mM EDTA, pH=8 | Clear cell renal cell carcinoma | - | 1:500 ON; RT | 1:250 ON;  4ºC | 1:100 ON; 4ºC |
| KDM3A | ab91252, abcam | 1:150 ON; RT | 1mM EDTA, pH=8 | Invasive urothelial carcinoma | 1:250 ON; RT | - | - | - |
| KDM6B | ab38113, abcam | 1:250 ON; RT | 1mM EDTA, pH=8 | Colon AC | 1:250 ON; RT | - | - | - |
| Ki-67 | M7240, DAKO | 1:150 ON; RT | Citrate 10mM, pH=6 | Breast cancer | - | - | - | - |
| KU-80 | 2180T, cell signaling | - | - | - | - | - | 1:1000 ON;4ºC | - |
| Mre11 | 4847T, cell signaling | - | - | - | - | - | 1:1000 ON;4ºC | - |
| NBS1 | 3001T (Ser343), cell signaling | - | - | - | - | - | 1:1000 ON;4ºC | - |
| RAD5O | 3427T, cell signaling | - | - | - | - | - | 1:1000 ON;4ºC | - |
| XLF | 2854T, cell signaling | - | - | - | - | - | 1:1000 ON;4ºC | - |
| Normal mouse IgG | 12-371, Millipore | - | - | - | - | - | - | 1:500 ON; 4ºC |
| RNA polymerase II | 05-623, Millipore | - | - | - | - | - | - | 1:500 ON; 4ºC |

AC, adenocarcinoma; IHC, immunohistochemistry; IF, immunofluorescence; ICC, immunocytochemistry; ON, overnight; RT, room temperature; WB, Western blot;
