# Supplementary figures and images for: JmjC-KDMs KDM3A and KDM6B modulate radioresistance under hypoxic conditions in esophageal squamous cell carcinoma
Source: Cell Death Dis. 2020 Dec 14;11(12):1068. doi: 10.1038/s41419-020-03279-y (PMC7736883; doi:10.1038/s41419-020-03279-y)

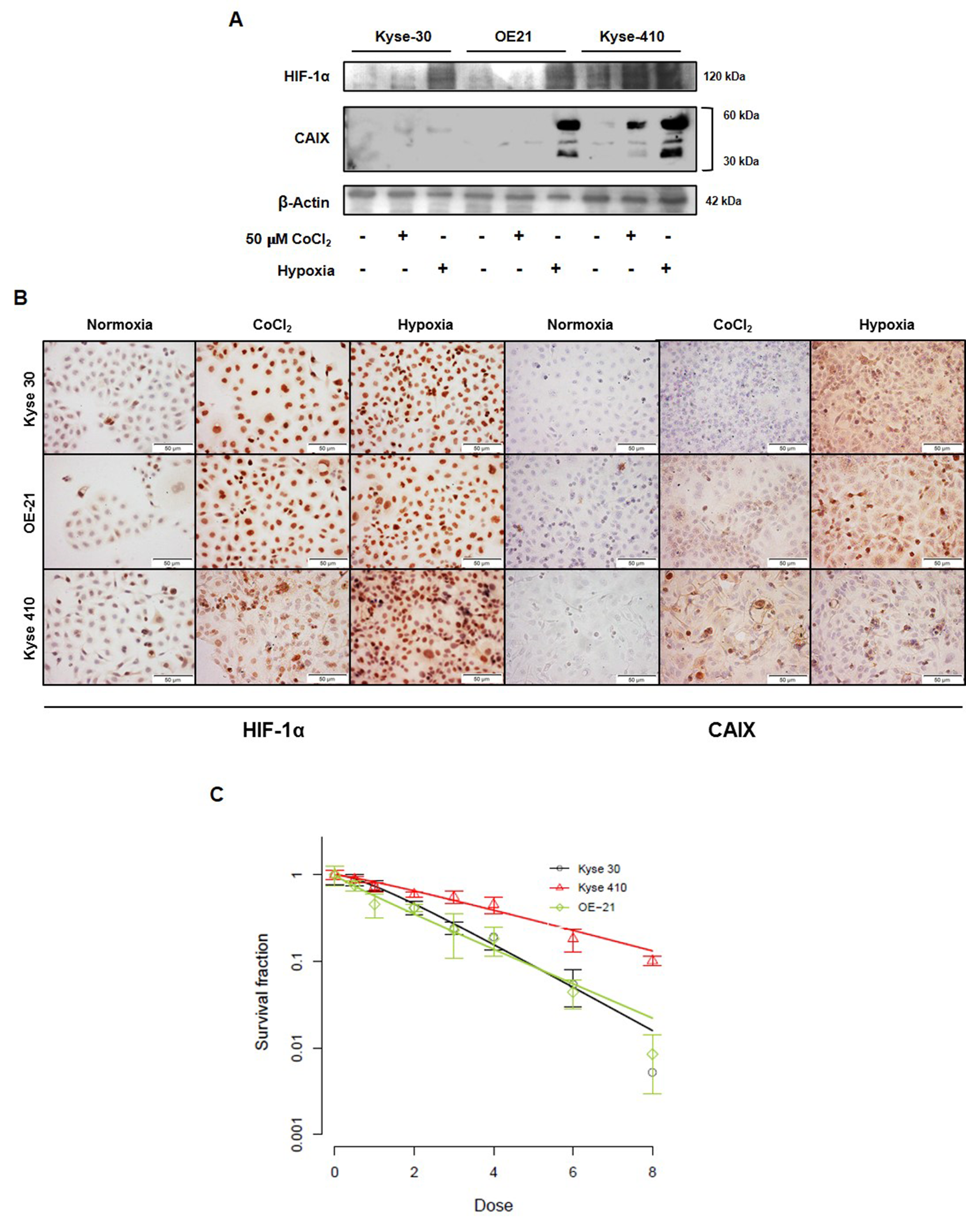

Supplement: Supplementary file 2 — Supplementary figure S1 [file 41419_2020_3279_MOESM2_ESM.tif]

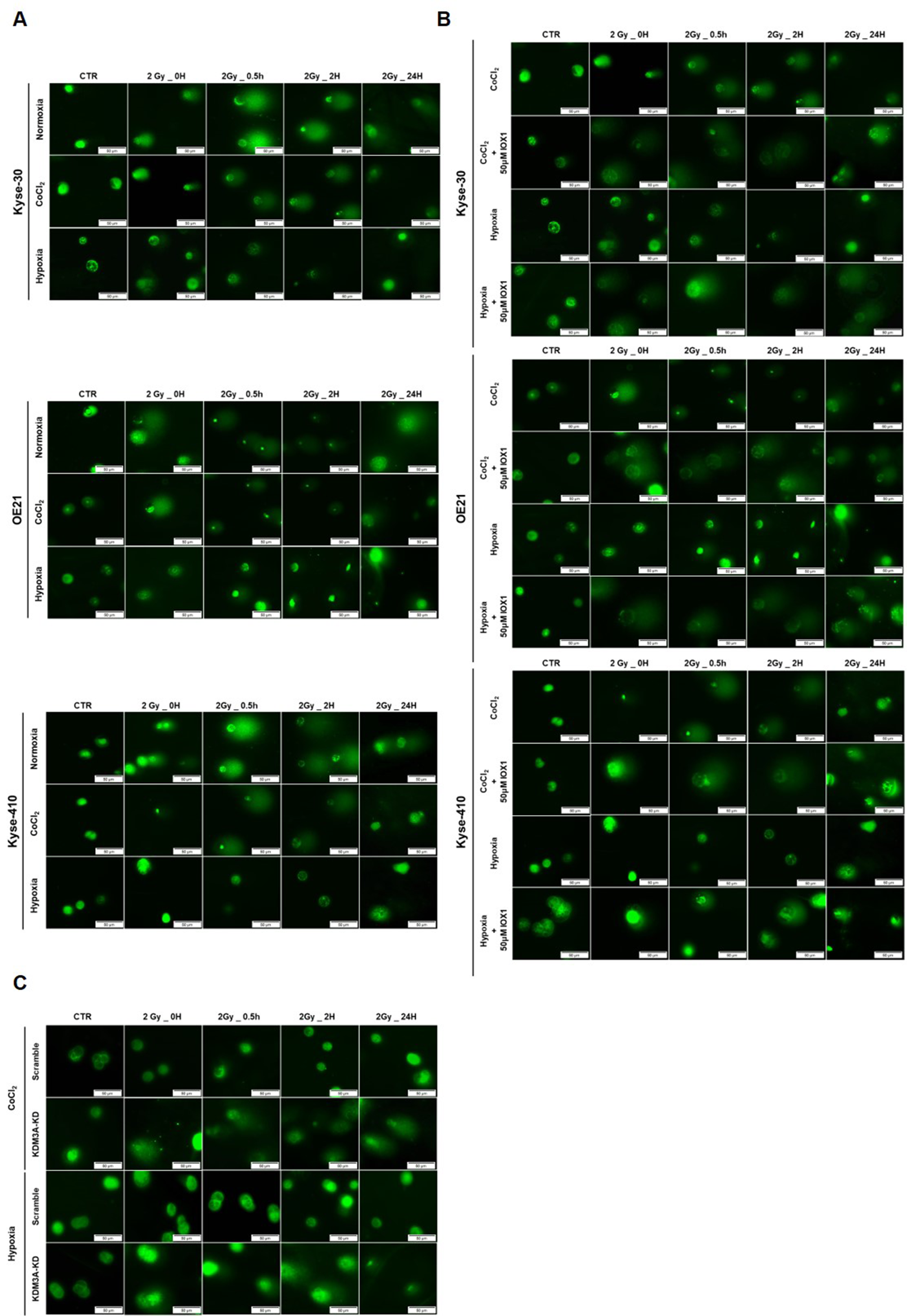

Supplement: Supplementary file 3 — Supplementary figure S2 [file 41419_2020_3279_MOESM3_ESM.tif]

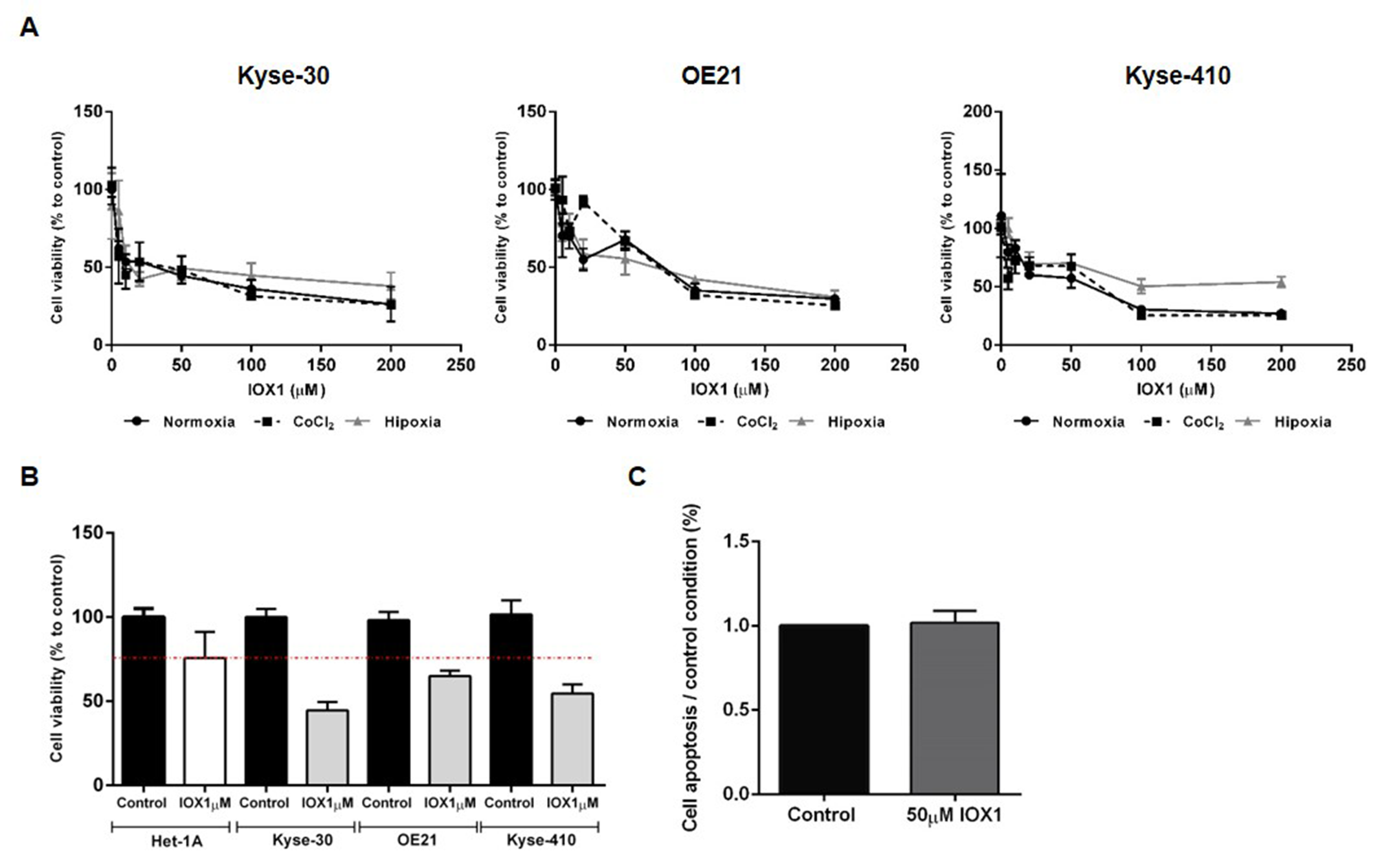

Supplement: Supplementary file 4 — Supplementary figure S3 [file 41419_2020_3279_MOESM4_ESM.tif]

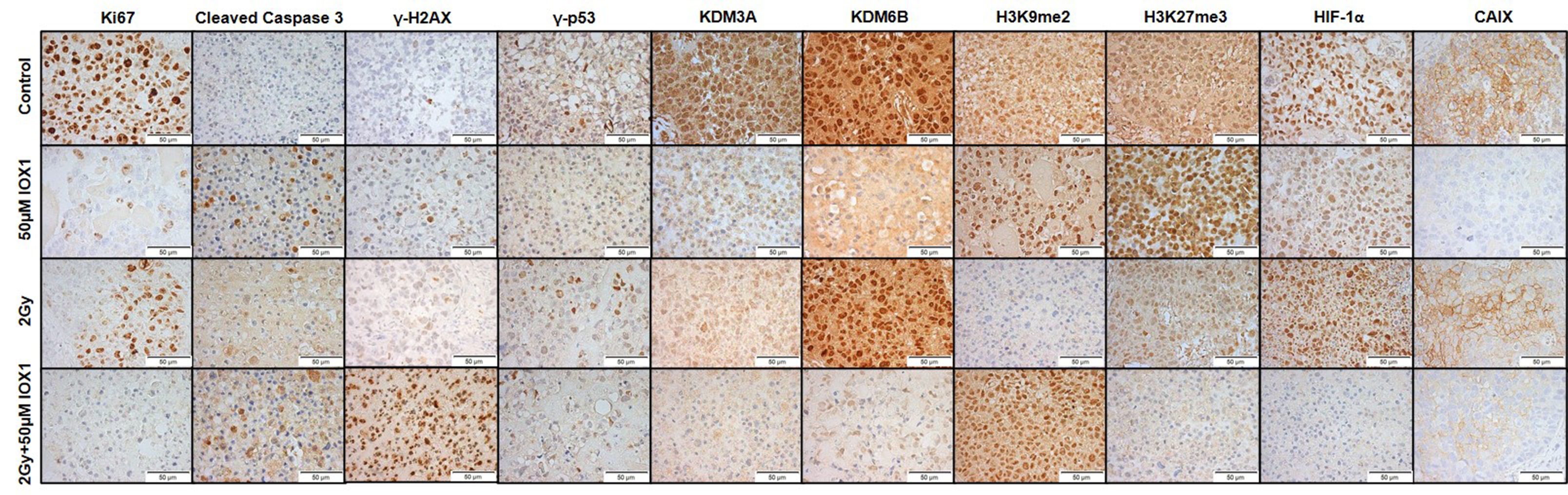

Supplement: Supplementary file 5 — Supplementary figure S4 [file 41419_2020_3279_MOESM5_ESM.tif]

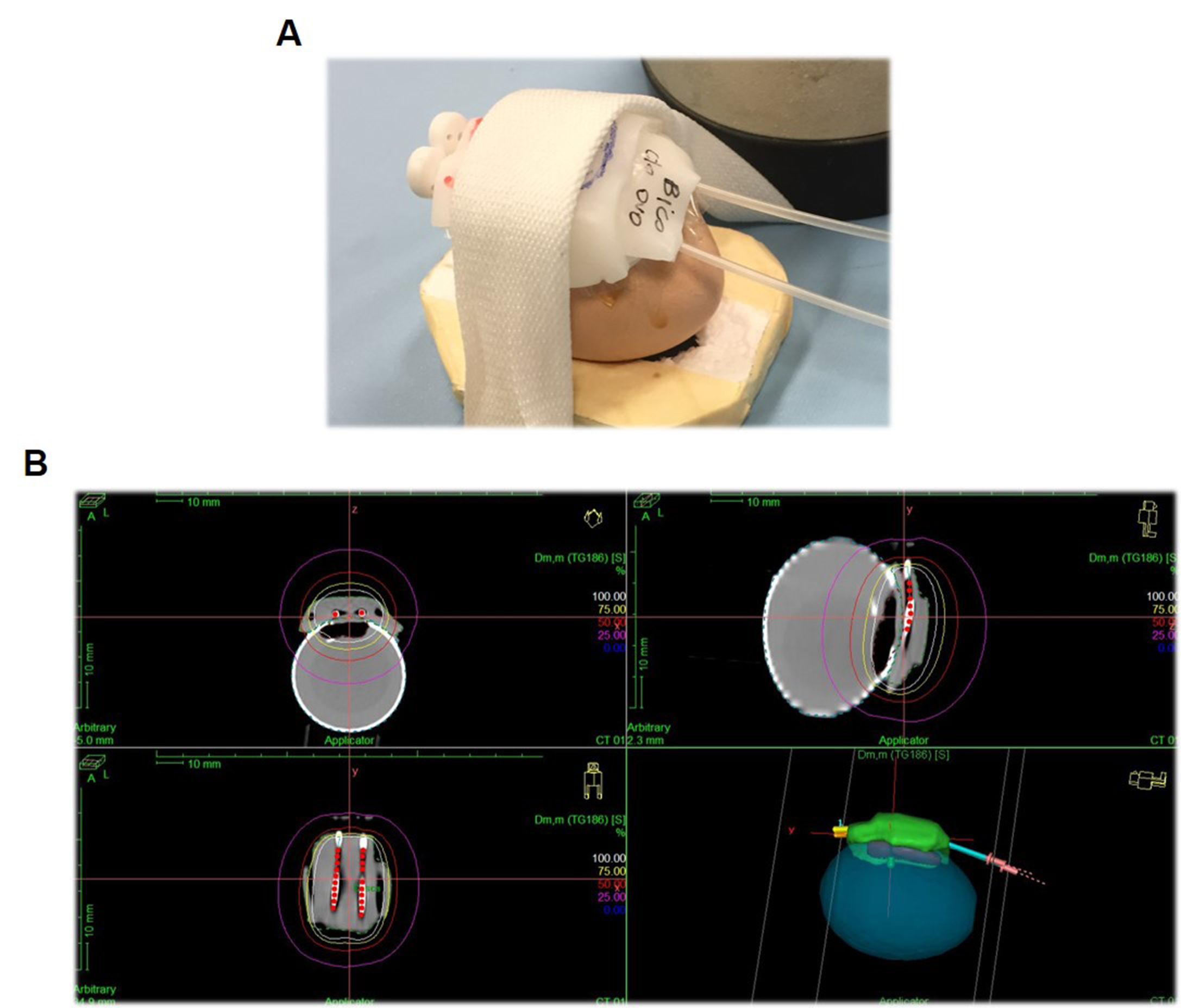

Supplement: Supplementary file 6 — Supplementary figure S5 [file 41419_2020_3279_MOESM6_ESM.tif]
