# Supplementary material for: JmjC-KDMs KDM3A and KDM6B modulate radioresistance under hypoxic conditions in esophageal squamous cell carcinoma
Source: Cell Death Dis. 2020 Dec 14;11(12):1068. doi: 10.1038/s41419-020-03279-y (PMC7736883; doi:10.1038/s41419-020-03279-y)
Supplement: Supplementary file 7 — Supplementary Figure Legends [file 41419_2020_3279_MOESM7_ESM.docx]

**Macedo-Silva et al_ Supplementary Figure Legends**

**Supplementary Figure S1. Hypoxic markers characterization in ESCC cell lines.** CAIX and HIF-1α protein expression in ESCC cell lines by Western blot **(A)** and immunohistochemistry **(B)** in normoxia, 50µM CoCl_2_ and hypoxia. Representative pictures were taken from Olympus IX51 microscope at 200x magnification (scale bar 50μm). **(C)** Cell survival curve of 3 ESCC cell lines, using SHMT model. Results are presented as mean ± SD of at least 3 independent experiments.

**Supplementary Figure S2. DNA damage after ionizing radiation in ESCC cell lines.** Representative images of nuclear DNA damage (SSB and DSD) **(A)** under normoxia, 50µM CoCl_2_ and hypoxia conditions **(B)** with 50µM IOX1 and **(C)** with KDM3A-KD/Scramble under 50µM CoCl_2_ and hypoxia conditions. All pictures were obtained with Olympus IX51 microscope at 200x magnification (scale bar 50μm).

**Supplementary Figure S3. Treatment of ESCC cell lines with IOX1 KDM inhibitor. (A)** Effect of IOX1 in cell viability of ESCC cell lines at normoxia, 50µM CoCl_2_ and hypoxia through MTT assay. Effect of 50µM IOX1 in cell viability **(B)** and cell apoptosis **(C)** of Het1-A cell line at normoxia condition.

**Supplementary Figure S4. ESCC microtumors characterization for cell proliferation, cell death and DNA damage.** Representative immunohistochemical pictures for Ki67, γ-p53, cleaved caspase 3, γ-H2AX, as well as KDM3A, KDM6B, H3K9me3 and H3K27me3 markers. Pictures were taken from Olympus IX51 microscope at 200x magnification (scale bar 50μm).

**Supplementary Figure S5. (A)** Representative image of an irradiated egg and **(B) 3D conformation treatment planning of** CAM assay with 2Gy irradiation in **microSelectronv3 Iridio-192 brachytherapy (192-Ir-mHDR-v2r). The four quadrants refer to the different treatment plan angles and incident areas of ionizing radiation that involves the tumor area in the chicken embryo chorioallantoic membrane. This specific zone was irradiated in a range of approximately 100% (white) of the planned dose. Further, in depth, there is a decrease in the absorbed dose to 75% (yellow), 50% (red) and 25% (purple), until no dose reached. In this way, the chicken embryo receives a minimal dose of radiation since it is located at the bottom of the egg, due to gravity.**
